# Clinical Trial Risk in Hepatitis C: Endpoint Selection and Drug Action

**DOI:** 10.1155/2016/6260271

**Published:** 2016-03-30

**Authors:** Nicole A. Tillie, Jayson L. Parker, Jordan J. Feld

**Affiliations:** ^1^Department of Biology, University of Toronto Mississauga, 3359 Mississauga Road, Mississauga, ON, Canada L5L 1C6; ^2^Toronto Centre for Liver Disease, Toronto General Hospital, 200 Elizabeth Street 9EN, Toronto, ON, Canada M5G 2C4

## Abstract

*Background and Aims.* This study analyzed the risk of clinical trial failure of new drugs for hepatitis C between January 1998 and January 2015.* Methods.* Hepatitis C drug development trials that were in phases I–III of clinical trial testing were obtained from the publicly accessible clinical trial repository and other publicly available databases. Drug compounds were excluded from the study if they began their phase I testing before 1998, if they were not industry sponsored, or if they treated secondary complications of hepatitis C. Clinical trial success rates were analyzed in comparison to industry expectations. Further analysis was conducted on the molecule classifications, the mechanisms of action, and the trial endpoints.* Results.* One hundred and twenty-three unique drug compounds were found to fulfill the inclusion criteria, eight of which had FDA approval. The overall cumulative pass rate for hepatitis C drugs was 20%, which is double the industry expectation rate. Viral inhibitor small molecule drugs significantly reduced the risk of drug failure during clinical trials compared to other mechanisms of action.* Conclusion.* On average, one in every five drugs that began clinical testing will be approved for market. Viral inhibitor small molecule drugs are the most promising and hold the least risk.

## 1. Background

The hepatitis C virus (HCV) has been recognized as a major cause of chronic liver disease since its discovery in 1989, chronically infecting between 130 and 150 million individuals worldwide [1, 2]. Based on a recent World Health Organization (WHO) study, there are between 350 000 and 500 000 deaths due to hepatitis C related liver diseases each year [2]. Fortunately, there are currently twelve drugs approved to treat hepatitis C, including four new drug approvals in the last 15 months, showing great promise in this field. Even with these new drug therapies, there are a much larger number of developing drugs that fail at various stages of clinical testing.

This retrospective study examined drug compounds that were in clinical testing between January 1998 and January 2015. The risk of developing a new drug for hepatitis C was quantified by comparing the cumulative pass rates for the new drug compounds against the previously reported industry expectations in order to highlight the factors that decreased such risk [3]. The factors influencing the risk of clinical trial failure were analyzed using similar methodology to the previous research investigating other disease areas [4–10].

## 2. Methods

### 2.1. Hepatitis C Study Eligibility and Study Patient Population

The methodology used to analyze the data in this paper has been previously employed to study the drug development risk for other disease indications [4–10]. The phase I, II, and III clinical trials for the treatment of hepatitis C were examined if they occurred between January 1998 and January 2015. A drug compound was excluded from analysis if the phase I testing began prior to 1998, if the trials were not industry sponsored, or if they were used to treat the secondary complications of HCV or HCV-HIV coinfections. The start date was selected based on the availability of the clinical data found through the website http://www.clinicaltrials.gov/. Prior to 1998, most data of the clinical trial failures is not publicly available.

### 2.2. Databases and Online Tools

The primary search tool used to collect the clinical trial data was the publicly accessible clinical trial repository available through the website http://www.clinicaltrials.gov/. Supplemental data were collected as needed through other publicly available databases. The search terms used were as follows: hepatitis C + phase 1, hepatitis C + phase 2, hepatitis C + phase 3, hepatitis C + approval, and hepatitis C + trials. Additional searches were conducted using the drug name in order to obtain specific information on the status and the fate of the drug.

### 2.3. Clinical Trial Outcome Classification

The classification of a successful drug varied depending on the clinical phase of the drug development. Phase I clinical trials were classified as a success if there was a phase II trial which was ongoing, recruiting, or has been completed. Similarly, phase II clinical trials were successful if they advanced to a phase III trial which was ongoing, was recruiting, or has been completed. Phase 3 clinical trials were classified as a success if they obtained US Food and Drug Administration (FDA) approval and the drug remained on the market. Additionally, a phase I/II trial was classified as a phase II trial and a phase II/III trial was classified as a phase III trial. FDA approval was chosen as the indication of a successful drug compound because the FDA is the regulatory agency for the world's largest market for pharmaceuticals [11].

A drug may have been classified as either a medical failure or a commercial failure. A medical failure indicates that the drug either had significant safety issues or failed to attain its primary endpoint. A commercial failure occurred if there was no further development of the drug for two or more years as seen on clinicaltrials.gov and press releases. Follow-up was completed until January 2015.

The clinical trial success rate was calculated by determining the percentage of unique drugs that successfully completed a phase of development out of the total number of drugs tested in a particular phase of development, as demonstrated using the following equation:

(1) $$\begin{array}{c} Transition\;\;probability\;\;for\;\;Phase\;\;x=\frac{\left(\#\;\;of\;\;drugs\;\;passed\;\;to\;\;Phase\;\;x+1\right)}{\left(\left(\#\;\;of\;\;drugs\;\;that\;\;passed\;\;to\;\;Phase\;\;x+1\right)+\left(\#\;\;drugs\;\;that\;\;failed\;\;at\;\;Phase\;\;x\right)\right)}. \end{array}$$

Drugs that were ongoing in Phase *x* were excluded in the transition rate for Phase *x*. The cumulative success rate refers to the probability of completing all of the phases of clinical trial testing (i.e., the product of the individual probabilities of success for each phase).

### 2.4. Endpoint Classification

Clinical trial endpoints were classified into two groups: the primary surrogate endpoint SVR12/SVR24 and other surrogate endpoints. Based on the FDA guidelines, the appropriate primary surrogate endpoint is SVR12—a sustained virologic response after 12 weeks of the completion of the treatment [12]. In prior years, the FDA recommended a sustained virologic response after 24 weeks (SVR24) as the surrogate endpoint [12]. Since many trials were conducted during that time frame, both SVR12 and SVR24 were accepted as the primary surrogate endpoint for this study. The other surrogate endpoints included changes from the baseline level of HCV RNA, early virologic response (EVR), rapid virologic response (RVR), SVR48, and SVR72, as well as normalization of alanine aminotransferase (ALT) levels.

### 2.5. Mechanism of Action and Molecule Classification

Drug compounds were classified based on their mechanism of action into four categories, which include immunotherapy, host inhibitors, viral inhibitors, and other. These mechanisms were categorized based on the drugs' specific targets as outlined by the manufacturer in press releases about the development of the drug. The drugs were later classified as small molecule drugs or biologics. Biologics include monoclonal antibodies, vaccines, and other drug therapies that were defined based on the FDA classification: “biological products are generally derived from living material—human, animal, or microorganism—and thus are usually not fully characterized” [13]. All compounds that were outside of the scope of this definition were classified as small molecule drugs.

## 3. Results

Using the search criteria listed in Section 2, 1950 clinical trial listings were found on clinicaltrails.gov, of which there were 123 unique drug compounds that satisfied the inclusion criteria. Drugs were included if their phase I testing began after January 1998 and if it was industry sponsored; they were excluded if they treated secondary complications of hepatitis C. To date, the FDA has approved twelve drugs for the treatment of hepatitis C but only eight of these began their phase I testing after January 1, 1998. The approved drugs include seven small molecule drugs, ribavirin (Rebetol, Merck & Co., USA), telaprevir (Incivek, Vertex Pharmaceuticals, USA), boceprevir (Victrelis, Merck & Co., USA), simeprevir (Olysio, Janssen Therapeutics, USA), sofosbuvir (Sovaldi, Gilead Sciences, USA), the combination of ledipasvir and sofosbuvir (Harvoni, Gilead Sciences, USA), and the combination of dasabuvir, ombitasvir, paritaprevir, and ritonavir (Holkira Pak, also known as Viekira Pak, AbbVie, USA), and one biologic: peginterferon alfa-2a (Pegasys, Genentech, USA) [14–21]. These drug compounds include six viral inhibitors, one immunotherapy drug, and one drug in which the mechanism of action has not been fully established. The latest approved drugs—Harvoni and Holkira Pak—are composed of several drug compounds, each with their own mechanism of action, and therefore each component was counted separately.

**Figure 1:**
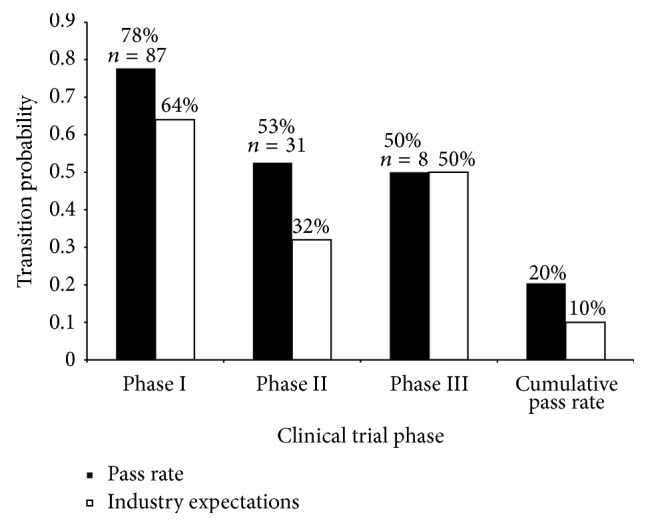
Clinical trial success rates. Clinical trial success rates in hepatitis C are compared against the industry expectations for each phase of clinical trial testing. The transition probability indicates the likelihood that a drug will successfully complete the phase of testing and will transition to the next phase or FDA approval for those in phase III. Cumulative pass rates represent the product of the probabilities of all three clinical trial phases. The sample size, *n*, indicates the number of drug compounds that have successfully completed that phase.

The clinical trial success rates for hepatitis C were analyzed in comparison to the industry expected success rates (Figure 1) [3]. The transition probability indicates the percentage of drug compounds in each clinical trial phase that were successful in transitioning to the next phase. The cumulative pass rate was calculated by the product of the transition probabilities of each phase of testing. This percentage indicates the probability that a drug will complete all of the clinical trial phases successfully. The cumulative pass rate is 20%, which is double the industry expectation of 10%, indicating that, overall, the drug development for hepatitis C exhibits promising outlook in terms of successful treatments. The success rates for phases I and II are exceeding the industry expectations with pass rates of 78% versus 64% and 53% versus 32% for phases I and II, respectively. The phase III success rate is equivalent to the industry expectations, both having a pass rate of 50%. The same analysis was conducted excluding all pegylated drug compounds, which resulted in similar results having pass rates of 77% for phase I, 51% for phase II, 47% for phase III, and 18% cumulatively. In all, drug development for hepatitis C tends to be slightly less risky than standard industry expectations indicate.

**Figure 2:**
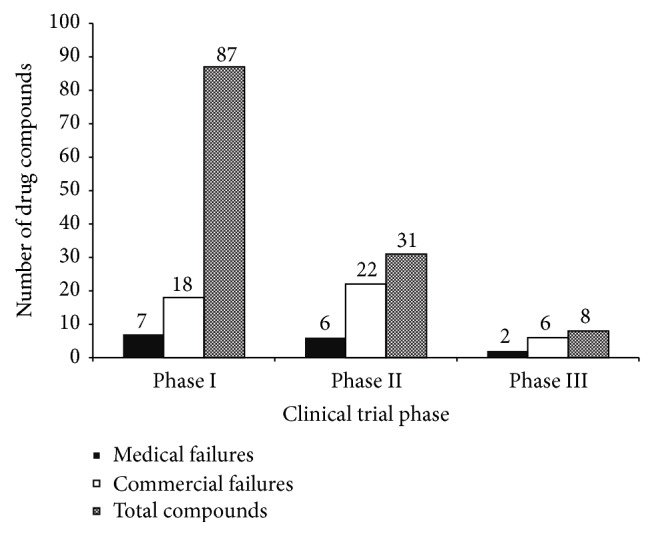
Clinical trial failures. The causes of clinical trial failures in the development of hepatitis C drugs are divided into medical failures and commercial failures. The number of drugs contributing to each type of failure in each clinical trial phase is shown. The total number of drug compounds that have passed the phase is depicted for reference.

Clinical trial failures were examined and divided into commercial and medical failures (Figure 2). In phases I and III, there was more than double the number of commercial failures as there were medical failures, whereas in phase II there was more than triple. Commercial failures are defined as no further development of a drug for at least two years. Of the 123 drugs being analyzed, only 15 had medical failures. Medical failures are significantly less frequent since they occur only once a drug has stopped development due to safety or efficacy issues. As the drug development continued, there were fewer incidences of medical failures and an increased number of commercial failures. The incidences of medical failures of the hepatitis C drugs included in this study were analyzed to determine if the improvement in preclinical testing resulted in fewer medical failures over the last decade, but the results showed no evident trend (data not shown).

**Figure 3:**
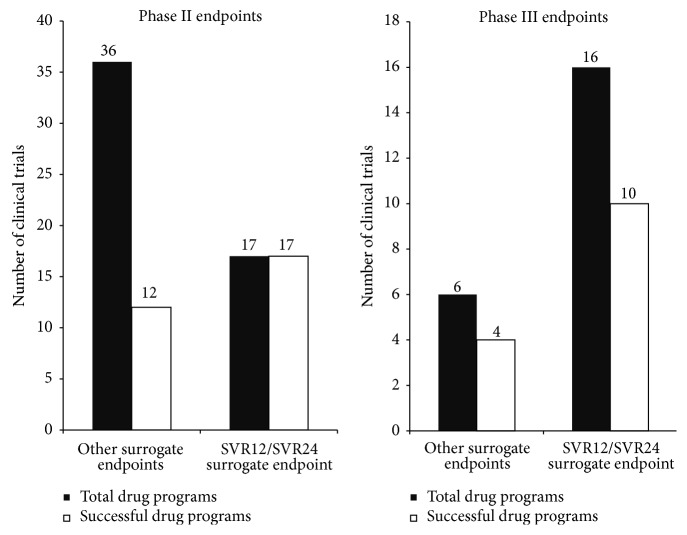
Endpoint selection. The surrogate endpoints for the phase II and III clinical trials were categorized based on the FDA recommended primary surrogate endpoint, SVR12/SVR24. Other surrogate endpoints included changes in baseline level of HCV RNA, EVR, RVR, SVR48, SVR72, and normalization of ALT levels. The success rates of drug trials with other surrogate endpoints versus those with the SVR12/SVR24 endpoints were contrasted by observing the total drug programs versus the successful programs.

The endpoints of the phase II and phase III clinical trials were analyzed based on the FDA guidance documents [12]. The endpoints were listed as either the primary surrogate endpoint SVR12/SVR24, a sustained virologic response after 12 or 24 weeks after treatment, which is the FDA recommended primary surrogate endpoint for these trials, or other surrogate endpoints, including early virologic response, rapid virologic response, SVR48, and SVR72 [12]. The phase II trials employed the other surrogate endpoints in many more trials, whereas the phase III trials favored the SVR12/SVR24 endpoint (Figure 3). The trials that had the SVR12 or SVR24 endpoint were very successful. There were no unsuccessful phase II trials that employed SVR12/SVR24 and nearly two-thirds of the phase III trials with this endpoint were successful, indicating that the SVR12/SVR24 endpoint provides less risk for trials in either phase.

**Figure 4:**
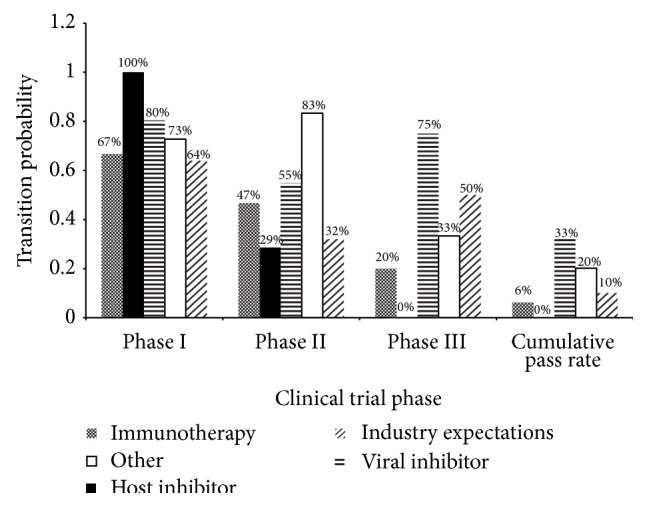
Success rate based on mechanism of action. The clinical trial success rates were further analyzed based on the mechanism of action of the drug compound. The four categories, immunotherapy, host inhibitors, viral inhibitors, and other, were compared against the industry expectation for the transition probability of that drug class. The cumulative pass rate indicates the overall probability that a drug from that class will be successful in all phases and achieve FDA approval.

The transition probabilities were calculated for the drugs in all three clinical trial phases based on the mechanism of action of each drug compound (Figure 4). The mechanisms of action were classified into four groups: immunotherapy, host inhibitors, viral inhibitors, and other. Host inhibitor drugs have yet to be approved by the FDA, although there are several drug candidates in clinical testing. Viral inhibitors appear to be the front-runners with the largest number of approved drugs and drugs in development. They have the highest cumulative pass rates of 33%, in comparison to the 10% industry expectation.

The specific drug targets that encompass each of the mechanisms of action were outlined and broken down into the number of small molecule drugs and biologics that employed these targets (Table 1). The favored targets included several of the HCV nonstructural proteins: NS5B polymerase, NS3/4A serine protease, and NS5A protein. These are the targets of six out of the eight FDA approved drugs and are mainly utilized by small molecule drugs, providing them with an advantage over biologics, which mainly favor other targets.

**Figure 5:**
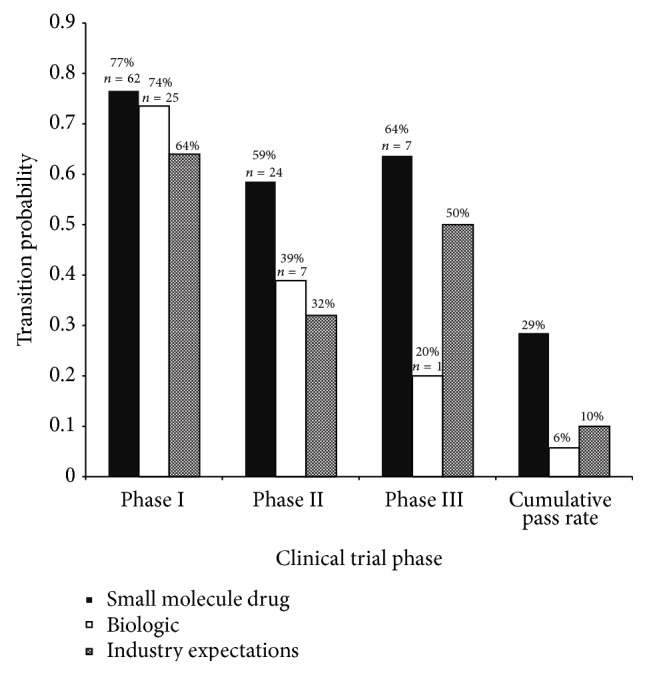
Success rate based on drug class. Clinical trial success rates in hepatitis C based on the drug class, being either a small molecule drug or a biologic. The transition probabilities for each phase as well as the cumulative pass rates were calculated. It is apparent that there are many more small molecule drugs in development and they have a much higher cumulative pass rate than biologics.

Finally, the drug classifications were analyzed and their transition probabilities were compared against the industry expectation rates (Figure 5). Seven of the eight FDA approved drugs are small molecule drugs, whereas only one is a biologic. Fewer than one-half of the developing drugs in each phase are biologics and therefore small molecule drugs have a significantly higher pass rate than biologics with a cumulative pass rate of 29% in comparison to 6% for biologics.

## 4. Discussion

Bringing new drugs to market is a challenge for all disease areas due to the lengthy process, the high cost of each phase, and the large number of clinical trial failures [12–22]. This study aimed to quantify the risk for clinical trials developing new hepatitis C drug therapies. The success rates and the factors influencing them were identified in this study in order to have a better understanding of the risk estimates associated with developing a new drug compound for this disease. The US Food and Drug Administration has approved 12 drugs to date that treat hepatitis C, eight of which satisfied our inclusion criteria [14–21]. By retrospectively screening clinicaltrials.gov from January 1998 to January 2015, the overall success rate for hepatitis C therapies was 20%, implying that on average one out of five drugs will make it to market. The drug chemistry, mechanisms of action, and targets were examined and the findings suggest that small molecule drugs that are viral inhibitors appear to carry the least risk. These drugs favor the HCV nonstructural proteins (NS3/4A, NS5A, and NS5B) as their targets, which are used significantly less frequently by biologics. Clinical trials that utilized the FDA recommended primary surrogate endpoint SVR12/SVR24 had substantially higher success rates over other surrogate endpoints [12]. This suggests that there are factors in each clinical trial that can reduce the risk of failure.

Although, as a whole, drug development for hepatitis C is much more successful than would be expected, there remain several factors that appear to reduce the risk of clinical trial failure. Small molecule drugs make up seven out of the eight FDA approved drugs that were analyzed in this study, and they also dominate in number over the biologics in development. Small molecules had a success rate nearly five times higher than that of the biologics, clearly demonstrating their superiority in this disease. This opposes several reports in the literature indicating the advantages and superiority of biologics [2, 4, 9, 10]. Although it appears that biologics are favored over small molecule drugs, there are also several studies indicating that small molecule drugs were more successful than biologics, implying that the drug chemistry that carries the least risk is dependent on the disease [5, 6]. For hepatitis C specifically, the tests and assays used in the development of the new drugs more accurately assess small molecule drugs and their targets; therefore these drugs are favored over biologics [23]. Small molecule drugs may be superior to biologics for hepatitis C simply based on what has previously been approved, but there are other factors, such as the drug targets as well as the available testing systems, that may have a larger impact on the outcome of the drug and that must be analyzed in conjunction with the drug chemistry.

After further analysis of the mechanisms of action and drug targets of the small molecule and biologic drug compounds, it is apparent that different targets are favored depending on the drug chemistry. The most prevalent drug targets were the HCV nonstructural proteins NS3/4A, NS5A, and NS5B, which are found in the most successful mechanisms of action—the viral inhibitors, which were heavily favored by the small molecule drugs. Although there are a few biologics that utilize these targets, the biologics' targets were found more frequently as immune adjuvants, monoclonal antibodies, toll-like receptor agonists, and interferons. It may therefore be the drug target as well as the available testing systems as opposed to the drug chemistry that is causing the higher success in the small molecule drugs. If this is the case, the biologics in development that utilize these HCV nonstructural proteins as targets may see more success in the future and may potentially result in more biologics being approved by the FDA.

The mechanism of action of the drugs is another key component contributing to the success of a drug therapy. Of the four mechanisms—immunotherapy, host inhibitors, viral inhibitors, and other—viral inhibitors are the most successful with a cumulative success rate of 33%. This mechanism directly targets the necessary components used in the progression and replication of the virus as opposed to immunotherapy, which increases the immune system defences, or other mechanisms in which the mechanisms are unclear or unknown. Since viral inhibitors target the structural and nonstructural proteins of the hepatitis C virus and they may prevent the function of certain receptors and enzymes needed in the development, growth, and replication of HCV, they show more substantial results in the reduction of HCV RNA, increasing the outcome of a sustained virologic response [24]. Although other mechanisms of action are useful in the drug therapy, it is the direct action of the viral inhibitors that prevents further replication and development of the viral genome, thus preventing the development of the disease [24].

Lastly, the endpoint selection of the phase II and III clinical trials provided an indication of the efficacy of the drugs in development. The FDA guidance document indicates that the recommended primary endpoint for developing antiviral hepatitis C drugs is the primary surrogate endpoint SVR12/SVR24—the sustained virologic response (viral RNA clearance) after 12 or 24 weeks after treatment [12]. All of the four drugs recently approved used this endpoint for both their phase II and phase III trials. In comparison, all of the drugs that failed phase II trials as well as one-quarter of the failed phase III drugs used other surrogate endpoints other than SVR12/SVR24. Surrogate endpoints provide an accelerated evaluation of the treatment since the primary outcome is not always easily measurable [25]. Every phase II drug that used the SVR12/SVR24 endpoint was successful, whereas the other surrogate endpoints for phase II only saw success for one-third of the drugs. In phase III, there was nearly three times the amount of drugs using the primary surrogate endpoint as opposed to another surrogate endpoint. Approximately 63% of drugs with the SVR12/SVR24 endpoint were successful, which was fairly similar to the success rate of the other surrogate endpoints. Similar results between the endpoints in phase III may be due to the small sample size in this phase in comparison to phase II. In all, the SVR12/SVR24 is a better predictor of the efficacy of the drug, as outlined by the FDA, and therefore more clinical trials should employ this as their primary endpoint [12].

There are some limitations to this study, which have been previously outlined in past research with similar methodology [4–10]. When categorizing the clinical trials into phase I, II, or III, the trials that had a combined phase (such as phase I/II or phase II/III) were classified as a trial of the latter phase. By categorizing the trial as the latter phase, this may have overestimated the success rates of the earlier phase. Some data may also appear to be inflated due to the small sample sizes for certain classifications.

## 5. Conclusion

The overall success rate of new drug development in hepatitis C therapies is 20%, which is double the industry expectation. Small molecule drugs, especially those that utilize the viral inhibitor mechanisms of action, appear to be the most promising and carry the least risk. The majority of the FDA approved drugs and those pending approval fall under these categories.

## Figures and Tables

**Table 1:** The number of small molecule and biologic drugs associated with known molecular targets.

Target	Small molecule	Biologic	Mechanism of action
HCV NS5B polymerase	32	3	Viral inhibitor
HCV NS3/4A serine protease	19	2	Viral inhibitor
HCV NS5A protein	17	1	Viral inhibitor
Mechanisms unclear/unknown	9	3	Other
Immune adjuvants	2	10	Immunotherapy
Viral replication inhibitor	2	2	Viral inhibitor
Toll-like receptor 9 agonist (TLR9)	0	4	Immunotherapy
Toll-like receptor 7 agonist (TLR7)	0	2	Immunotherapy
Cyclophilin	2	0	Host inhibitor
Blocks viral entry	2	0	Host inhibitor
Caspase inhibitor	2	0	Host inhibitor
Interferon	0	6	Immunotherapy
HCV internal ribosome entry site (IRES) in 5′UTR	1	1	Viral inhibitor
Inosine monophosphate dehydrogenase	1	0	Host inhibitor
Alpha-glucosidase 1	1	0	Host inhibitor
Monoclonal antibodies	0	5	Immunotherapy
Mature miR-122 (a liver microRNA)	0	1	Host inhibitor
